# Editorial: 365 days of progress in radiation oncology

**DOI:** 10.3389/fonc.2023.1179316

**Published:** 2023-03-21

**Authors:** Timothy J. Kinsella

**Affiliations:** Department of Radiation Oncology, Warren Alpert Medical School, Brown University, Providence, RI, United States

**Keywords:** FLASH RT, glutamine metabolism inhibitors + RT, volumetric modulated arc radiotherapy, hydrogel based biosensors, RT induced eosinophils

This wide-ranging Frontiers in Radiation Oncology (FRO) Research Topic for 2022 attempted to highlight some recent, but continuously evolving, biologic and technical concepts to maximize the therapeutic gain in Radiation Oncology in tumor control with minimal (ideally none) toxicity to normal tissues. While very ambitious in scope, 6 FRO manuscripts were published and are summarized as follows.

Two articles, including a review by Lin et al. from Mianyang Central Hospital and the Institute of Applied Electronics of the China Academy of Engineering as well as a clinical article by Calvo et al. from Madrid, Spain and Orsay, France focus on the concept and potential of FLASH radiotherapy (RT). FLASH RT delivers ultra-high dose rates (>40 Gy/sec) which are 300-400 X shorter than conventional RT. Limited pre-clinical studies using photons, electrons and protons have documented relative sparing of RT damage to various types of normal tissues with FLASH RT with similar tumor control compared to conventional RT. Given the reduced RT associated normal tissue toxicities with FLASH RT, it’s future use may allow for significantly higher RT doses in clinically radioresistant human cancers such as high grade gliomas and sarcomas. In their recently published review the authors expand on their prior FLASH RT review to better explain the physical-chemical interactions and biological mechanisms including altered DNA repair, oxygen depletion, immune function and blood vessel effects. Unfortunately, the current published data cannot fully explain the FLASH RT effect.

The second Research Topic publication by Calvo et al. argues that a dual FLASH RT approach using both proton and intraoperative electron beam RT (IOERT) will be of value in patients with oligo-metastatic, locally recurrent and locally unresectable cancers. They detail their multi-disciplinary approach in 5 patients using today’s conventional proton and IOERT RT treatments, where with further FLASH RT technologies, there should be an increase in the therapeutic gain. A recently published abstract from the 2022 ASTRO conference dealing with a small clinical trial (FAST-01; NCT 04592887; https://www.astro.org/News-and-Publications/News-and-Media-Center/News-Releases/2022/FLASH-radiation-therapy-shows-promise-in-first-in) of FLASH proton RT to alleviate pain in bone cancers supports this approach.

A third paper published under this 2022 FRO Research Topic involves a mini-review of novel chemical modifiers of glutamine metabolism as potential radiosensitizers in pre-clinical human cancer models. Alden et al. from SUNY Upstate Medical University provide an overview of glutamine metabolism as it relates to ionizing radiation resistance focusing on effects on DNA repair and anti-cancer immunity based on recent literature. Additionally, they highlight the “bench-to-bedside” on-going clinical trials of specific glutamine metabolism inhibitors and radiation therapy in IDH-mutant gliomas and locally advanced cervix cancer based on the pre-clinical literature.

The fourth Research Topic paper by Chen et al. from Xiangya Hospital in China involves a radiation therapy treatment and clinical outcomes study in patients with non-metastatic, late T-stage nasopharyngeal cancer. They compare outcomes using volumetric modulated arc therapy (VMAT) versus tomotherapy. Such patients present major challenges for modern day radiation therapy treatment planning because of the primary tumor location and multiple loco-regional normal tissues. Using a 1:1 propensity score match of 171 patients in this single institution study, the authors demonstrate that tomotherapy is superior to VMAT in terms of most normal tissue (or organs at risk, OAR) dosimetric parameters with a decrease in acute mucositis (Grade ≥ 3; 22 vs 41%; p=0.038) and a higher clinical complete response (CR= 83 vs 67%; p=0.046). No differences in local control nor overall survival were found. However, these authors also suggest that a hybrid radiation treatment planning combining intensity-modulated radiation therapy (IMRT) and VMAT in locally advanced nasopharyngeal carcinoma may be equivalent or superior to tomotherapy in terms of toxicities and clinical outcome.

The fifth paper involves a review article by Xie et al. which provides an update of hydrogel-based local drug/biologic/radioisotope delivery systems to enhance post-operative radiation therapy. Hydrogels are a new class of local drug/biologic/radioisotope delivery with theoretically provide 3 major advantages compared to systemic delivery including biocompatibility, high loading capacity and sustained local drug release. They describe different classifications of hydrogels based on physical material, cross-linking patterns, electric charge and polymeric composition. They illustrate pre-clinical use of hydrogels for local delivery of radioisotopes including I^125^, I^131^ and Rh^188^ as well as immune modulators including PD-L1 antibodies. They also cite the complexity in synthesis, delivery and local toxicities of the hydrogel delivery systems.

The final paper in this FRO Research Topic by Wang et al. describes the impact of changes in absolute eosinophil counts measured weekly during and for one month following definitive radiation therapy (> 46 GY total dose) as a clinical biomarker predicting overall survival (OS) and progression free survival (PFS) in patients with locally advanced non-small cell lung cancer (NSCLC). In a retrospective, single institution review of 240 patients from Chongqing University in China, an eosinophil increase ration (EIR) of ≥ 1.43 at 1 month post-treatment was predictive of favorable clinical outcomes (5 year OS: 21% vs 10%, p<0.001 and 5 year PFS: 10% vs 8%, p=0.014). The use of concomitant versus sequential chemotherapy did not impact PFS. Interestingly, the heart mean dose and heart V10 were directly associated with a lower EIR but did not impact OS and PFS in this retrospective review. Additionally, no analysis of key molecular markers (EGFR, ROS1) nor immune status (PD-1, PD-L1) were part of this retrospective review.

In summary, I highlight the key factors from the 6 manuscripts published under the wide-ranging FRO Research Topic, “*365 Days of Progress in Radiation Oncology*.” As expected, there are no overall take-home messages nor common lessons from these manuscripts. Hopefully, they provide a ground base for future basic and clinical research in radiation oncology.

## Author contributions

The author confirms being the sole contributor of this work and has approved it for publication.

